# Outcome of Intra-articular Injection of Total Stromal Cells and Platelet-Rich Plasma in Primary Knee Osteoarthritis: A Randomized Clinical Trial

**DOI:** 10.7759/cureus.34595

**Published:** 2023-02-03

**Authors:** Moshiur R Khasru, Md. Abu B Siddiq, Talukder AGM Zakaria N Jubery, Tangila Marzen, Ashraful Hoque, AKM Zahir Ahmed, Masuda Begum, Fazle R Chowdhury, Abul Khair M Salek, Md. Moniruzzaman Khan

**Affiliations:** 1 Physical Medicine and Rehabilitation, Bangabandhu Sheikh Mujib Medical University, Dhaka, BGD; 2 Physical Medicine and Rehabilitation, Brahmanbaria Medical College, Brahmanbaria, BGD; 3 Rheumatology, University of South Wales, Pontypridd, Wales, GBR; 4 Burn and Plastic Surgery, Dhaka Medical College Hospital, Dhaka, BGD; 5 Anatomy, Shaheed Suhrawardy Medical College Hospital, Dhaka, BGD; 6 Blood Transfusion, Sheikh Hasina National Institute of Burn & Plastic Surgery, Dhaka, BGD; 7 Musculoskeletal Sonography, Module General Hospital, Dhaka, BGD; 8 Hematology, Bangabandhu Sheikh Mujib Medical University, Dhaka, BGD; 9 Internal Medicine, Bangabandhu Sheikh Mujib Medical University, Dhaka, BGD

**Keywords:** mesenchymal stem cell, total-stromal-cells (tost), single intra-articular injection, knee osteoarthritis, osteoarthritis

## Abstract

Introduction: Mesenchymal stem cell (MSC) therapy appeared promising in knee osteoarthritis (OA). We examined if a single intra-articular (IA) autologous total stromal cells (TSC) and platelet-rich plasma (PRP) injection improved knee pain, physical function, and articular cartilage thickness in knee OA.

Methods: The study was performed in the physical medicine and rehabilitation department of Bangabandhu Shaikh Mujib Medical University, Dhaka, Bangladesh. Knee OA was diagnosed according to the American College of Rheumatology criteria and randomly assigned to treatment (received TSC and PRP) and control groups. Kallgreen-Lawrance (KL) scoring system was used to grade primary knee OA. The Visual Analogue Scale (VAS, 0-10 cm) for pain, WOMAC (Western Ontario and McMaster Universities Arthritis Index) for physical function, and medial femoral condylar cartilage (MFC) thickness (millimeters) under ultrasonogram (US) were documented and compared between groups before and after treatment. Statistical Package analyzed data for Social Scientists (SPSS 22.0; IBM Corp, Armonk, NY) was used for data analysis. Pre- and post-intervention outcomes were measured using the Wilcoxon-sign test, whereas Mann-Whitney U-test calculated the difference between groups; a p-value <0.05 was considered statistically significant.

Result: In the treatment group, 15 received IA-TSC and PRP preparation, and in the control group, 15 patients received no injection, but quadricep muscle-strengthening exercise. There was no significant difference between groups regarding VAS for pain, WOMAC physical function, and cartilage thickness before starting the treatment and two weeks after intervention. VAS for pain and WOMAC physical function scores improved profoundly in the treatment group after 12 and 24 weeks of intervention; the pain and physical function scores difference between groups was also significant. However, significant mean femoral cartilage thickness was not changed until the end of 24 weeks (U=175.00, p=0.009 two-tailed and U= 130.00, p=0.016 two-tailed, respectively, for right and left knee).

Conclusion: Single TSC and PRP injection reduces knee pain and improves physical function and cartilage thickness in knee OA. While pain and physical function improvement happen earlier, cartilage thickness change takes more time.

## Introduction

Osteoarthritis (OA) of the knee is the most prevalent degenerative joint disease, with gradual loss of articular cartilage volume and thickness [1]. Primarily, it affects the elderly, with a global prevalence of over 23%; more than 654 million cases were estimated in 2020 for ages over 40 years [1,2]. Knee OA prevalence measured highest (10.6%) in affluent-urban communities and lowest in rural dwellers [3]. Cartilage degeneration is the hallmark of OA [1]. Nowadays, knee OA is considered a total joint disease; besides articular cartilage degeneration, inflammatory synovitis, subchondral bone remodeling (thickening, bone collapse, bone cysts), meniscal degeneration-tear-extrusion, and capsular hypertrophy are also seen in the disorder [4].

OA knee has some modifiable and non-modifiable risk factors [1,2,5]. OA knee presents with joint pain, swelling, limited range of motion (ROM), deformities, and impaired daily activities. Increased sick leave, decreased mobility, associated depression, and joint replacement, especially in advanced cases, contribute to significant socioeconomic burdens and healthcare costs [6,7]. So early diagnosis is crucial.

Management of OA involves pharmacological and non-pharmacological interventions [1,8]. Recommended non-pharmacological treatment includes proper activities of daily living (ADLs) instructions, therapeutic exercise for the knee, weight reduction, cognitive behavioral therapy, tai chi, orthoses, braces, and acupuncture [1]. Oral non-steroidal anti-inflammatory drugs (NSAIDs), paracetamol, intra-articular (IA) steroids, injectable viscosupplementation (hyaluronic acid [HA]), neutraceuticals (glucosamine and chondroitin sulfate), duloxetine, tramadol, topical NSAIDs, and capsaicin provide short-time pain relief [1,8]. Surgical options are reserved for severe OA knee cases [1]. Unlike autoimmune inflammatory arthritis, disease-modifying agents are not helpful in knee OA [4,9].

Growing evidence suggests that platelet-rich plasma (PRP) and mesenchymal stem cell (MSC) therapy induce cartilage regeneration [10,11]. Adipose tissue-derived MSC (ADMSC) and peripheral blood stem cells can reduce knee pain and improve physical function and overall cartilage quality in OA [12,13]. In a recent meta-analysis, the ADMSC-PRP combination showed good clinical efficacy in terms of pain reduction and improving joint function in knee OA [14]. However, further research must address research bias, long-term outcomes, uniform PRP, and MSC preparation technique.

Obtaining regenerative cells from adipose tissue can be done in two ways: enzymatic and mechanical. ADMSC-derived stromal vascular fracture is found in the enzymatic method. In contrast, total stromal cells (TSC) consisting of stromal cells, vascular endothelial growth factor A, epithelial growth factor A, fibroblast growth factor 2, platelet-derived growth factor, nerve growth factor, and transforming growth factor-β1 are found in a less expensive mechanical method that protects the stem-cell microenvironment, extracellular matrix, and regenerative cells [15].

In this randomized controlled trial (RCT), we aimed to see how a single IA TSC and PRP injections affected knee pain, overall physical function, and articular cartilage thickness in OA knee. Since we are yet to find a cure for cartilage degeneration, simplistic procedures would be hope for the disorder.

## Materials and methods

This single-blind RCT was performed with primary knee OA according to the American College of Rheumatology criteria [16] attended in the department of physical medicine and rehabilitation, Bangabandhu Sheikh Mujib Medical University (BSMMU), between March 2018 and December 2021. Kellgren-Lawrence (KL) radiological scores were used to define OA [16]. The ethical committee approved the clinical trial (Memo No. BSMMU/2018/25) and registered with www.clinicaltrials.gov (Identifiers NCT05280002). Informed written consent was taken from all study participants.

Study participants

Primary OA knee with KL-grade II and III radiological changes were enrolled. Patients treated with IA steroid, viscosupplementation, PRP, or knee surgery within the last six months were excluded. We excluded septic and tubercular arthritis, post-traumatic hemarthrosis, unstable knee joint due to an anterior cruciate ligament injury, malignancy, crystal-induced arthritis, rheumatoid arthritis, psoriatic arthritis, reactive and ankylosing spondylitis, and Charcot arthropathy. Patients with local eczematous lesions and cellulitis were exempted from the research.

Ultrasonogram (US) examination 

The patient was positioned supine with about 30-degree knee flexion with a supported knee back pillow. Sweeping of the probe over the anterior and medial knee was done; the knee flexed as much as possible while measuring the medial femoral condylar cartilage (MFC) thickness. Podlipska et al. described US-depicted qualitative MFC thickness [17]: Grade 0 - a monotonous anechoic band with sharp hyperechoic anterior and posterior interfaces, Grade 1 - loss of the average sharpness of cartilage interfaces and/or increased echogenicity of the cartilage, Grade 2a - grade 1 plus thinning of articular cartilage less than 50%, Grade 2b - more than 50% but less than 100% thinning of articular cartilage, Grade 3 - 100% local loss of the cartilage tissue. However, a quantitative MFC thickness of less than 2.2 mm was considered cartilage thinning [17].

Randomization of study subjects and interventions**

A total of 30 OA was randomly (simple random sampling) divided into treatment and control groups. Fifteen patients in the treatment groups received a single dose of 6 milliliters (ml) TSC and 3 ml PRP. PRP was activated with 0.1 ml calcium gluconate per 0.9 ml PRP (1:9 ratio); controls did not receive TSC or PRP injection. Both groups received acetaminophen 1 g thrice daily for 14 days for pain, isometric quadriceps muscle exercise, aerobic exercise, mind-body exercise, and ADLs instructions [1,4]. Superficial heating was applied in control, where appropriate. The patients were assessed at week 2, at 12 and 24 weeks after the intervention.

Preparation and IA injection of TSC and PRP

**Figure 1:**
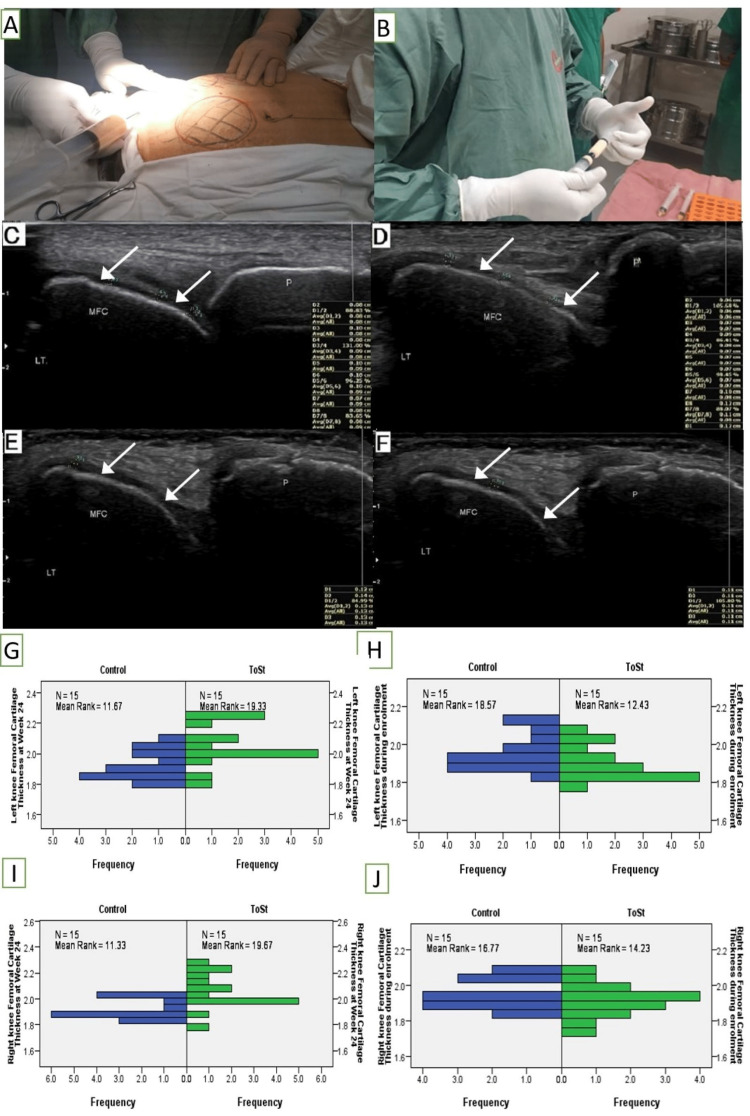
TSC preparation steps, and cartilage thickness measures before and after intervention (A, B) Lipo-aspiration and processing for TSC; (C, D) medial femoral condylar cartilage thickness before TSC; (E, F) cartilage thickness after TSC injection; (G-J) cartilage thickness measures in right and left knee before and after TSC injection. Arrows point to articular cartilage. TSC, total stromal cells; P, patella; MFC, medial femoral condylar cartilage; LT, left knee.

In the daycare procedure room, 50 ml of packed adipose tissue was obtained by liposuction of the subcutaneous layer of the lower abdominal area using manual techniques [13]. After cleaning the lower abdominal area with 10% betadine, the patient was draped using a sterile fashion. Using the tumescent solution, the lower abdomen was locally anesthetized. Then, the tumescent solution was infiltrated into subcutaneous adipose tissue using a filtration cannula. Afterward, using a 3.0 cannula connected to a 60-mL Luer-Lock syringe, 50 ml of adipose tissue was obtained (Figure 1A, 1B).

A skilled plastic surgeon did liposuction. The aspirated fat was processed by a trained regenerative physiatrist (MRK). These lipo-aspirates were then kept upright for 10 minutes to separate adipose tissue from blood and oil. Collagenase was mixed for easy breakdown of the complex fat. The packed adipose tissue was then transferred back to the 5 ml disposable for manual tissue homogenizer using 2.4, 1.4, and 1.2 sized transfer adaptors for 30 repetitions each resulting in cutting and mincing the adipose tissue. After that, the processed adipose tissue was filtrated using nano-filter transfer. Finally, 6 ml of TSC was transferred in a 10 ml disposable syringe to mix with activated PRP for IA injection in each knee. Simultaneously, 2 ml of TSC [15] from each procedure was sent to a laboratory for stem cell markers. The sample was positive for MSC markers CD44 and CD90 and negative for hematopoietic stem cell markers DC34 and CD45.

Venous blood was initially collected in tubes that contain anticoagulants [18]. PRP was prepared from venous blood using a double centrifugation technique by a transfusion medicine expert. Initial centrifugation separates red blood cells (RBCs); the second centrifugation concentrates platelets suspended in the smallest final plasma volume [18]. After the first spin step, the venous blood separates into three layers: an upper layer mainly containing platelets and white blood cells (WBCs), a thin intermediate layer known as the buffy coat and rich in WBCs, and a bottom layer that consists primarily of RBCs. The upper layer and superficial buffy coat were transferred to an empty sterile tube for pure PRP production. The second spin step was then performed. Pellets were homogenized in the lower one-third (5 ml of plasma) to create the PRP [18]. A baseline blood cell count was done and compared with that of the PRP. At least a threefold rise in platelet count was considered a satisfactory preparation of PRP. Calcium gluconate was used to activate PRP [18]. IA injection of TSC and PRP was performed in a flexed knee joint with a superolateral approach [19]. An expert having expertise in knee interventions performed the interventions. 

Outcome measures

A prefabricated questionnaire was used for data collection. Visual analog scale (VAS, 1-10 cm) for pain, WOMAC for pain and physical function, and MFC cartilage thickness were the outcome variables. MFC thickness measurements were taken in maximum knee flexion [17]. WOMAC is a 24-item self-report questionnaire that includes domains for pain (five parts), stiffness (two fields), and physical function (17 parts) [20]. All scores were converted on a scale of 0-100 for better representation. VAS for pain, WOMAC for pain and physical function, and MFC thickness were measured before and six months after TSC and PRP intervention. Figure 1 demonstrates TSC preparation steps and cartilage thickness changes after the intervention (Figure 1C-1J). 

Immediate post-procedural events, namely, injection site bleeding and vasovagal shock, were observed for 30 minutes and documented if seen. The patients were advised relative rest for the next 72 hours to allow adequate time for adherence to the stem cells with the cartilage margin. Post-procedural pain was managed with paracetamol and tramadol. All the respondents were solicited to visit the clinic for scheduled follow-ups. The patients were kept in touch over the telephone weekly and encouraged to continue therapeutic exercises and follow ADL instructions.

Statistics and data analysis

Data were compiled and sorted correctly and analyzed statistically using Statistical Package for Social Scientists (SPSS, version 22.0; IBM Corp., Armonk, NY). Qualitative and quantitative variables were expressed as percentages and mean ± standard deviation (SD), respectively. Comparisons of VAS for pain (0-10 cm), WOMAC physical function, and MFC thickness scores were documented before and after the interventions and also between groups. Pre- and post-intervention outcomes were measured using the Wilcoxon-sign test. Mann-Whitney U-test calculated the difference between groups, and p-value <0.05 at a 95% confidence interval was considered statistically significant. Tables and graphs are used to present data.

## Results

Table 1 represents the demographic profile of the study participants.

**Table 1:** Demographic profile of study participants TSC, total stromal cells; VAS, visual analog scale; WOMAC, Western Ontario and McMaster University Osteoarthritis Index; KL, Kallgreen-Lawrance; MFC, medial femoral condylar cartilage; n, frequency number; %, percentage; SD, standard deviation. ^a^p-Value obtained from chi-square test; ^b^p-Value obtained from Fisher’s exact test.

Particulars		TSC (n, %)	Control (n, %)	p-Value
Gender	Female	9, 60%	10, 66.67%	0.705^a^
Age in years	Mean±SD	63.20±5.36	64.27±6.29	-
BMI (kg/m^2^)	Mean±SD	26.75±2.44	27.50±2.31	-
	Normal weight	5, 33.33%	2, 13.33%	0.231^b^
	Overweight	10, 66.67%	11, 73.33%	
	Obese	0, 0%	2, 13.33%	
K-L grading	Grade II	6, 40%	5, 33.33%	0.705^a^
	Grade III	9, 60%	10, 66.67%	
Comorbidities	Diabetes	6, 40%	5, 33.33%	0.744^b^
	Hypertension	4, 26.67%	5, 33.33%	
	Brachial asthma or COPD	2, 13.33%	0, 0%	
	More than one comorbidity	2, 13.33%	4, 26.67%	
	None	1, 6.67%	1, 6.67%	
Pain severity	No pain	0	0	
	Mild	0	0	
	Moderate	14	15	
	Severe	1	0	
VAS, pain	Right	5.63±0.67	5.67±0.49	0.50^b^
	Left	5.73±0.70	5.53±0.74	0.388^b^
WOMAC physical function		39.47±6.21	37.60±6.29	0.390^b^
	Mild limitation	0	0	
	Moderate limitation	2	5	
	Severe limitation	13	10	
MFC cartilage thickness	Right	1.92±0.10	1.96±0.08	0.254
	Left	1.90±0.09	1.96±0.09	0.085

Pre-intervention pain intensity (VAS, 0-10 cm) and WOMAC physical function scores were measured and compared with post-intervention measures in the TSC group in Tables 2, 3, respectively. 

**Table 2:** Comparison of VAS pain and MFC thickness before and after TSC and PRP injections MFC, medial femoral condylar cartilage thickness; VAS, visual analog scale; z, test statistics. p-Value was obtained from Wilcoxon signed rank test.

Particulars			N	Mean rank	Sum of ranks	z-Value	p-Value
VAS (0-10 cm) pain: 2 weeks after treatment	Right knee	Negative rank	29	16	464.00	-4.832	0.000
		Positive rank	1	1	1.00		
	Left knee	Negative rank	29	16.00	464.00	-4.843	0.000
		Positive rank	1	1.00	1.00		
VAS (0-10 cm) pain: 12 weeks after treatment	Right knee	Negative rank	29	15.00	435.00	-4.749	0.000
		Positive rank	0	0.00	0.00		
	Left knee	Negative rank	29	15.00	435.00	-4.732	0.000
		Positive rank	0	0.00	0.00		
VAS (0-10 cm) pain: 24 weeks after treatment	Right knee	Negative rank	28	14.50	406.00	-4.655	0.000
		Positive rank	0	0.00	0.00		
	Left knee	Negative rank	28	14.50	406.00	-4.646	0.000
		Positive rank	0	0.00	0.00		
MFC thickness 24 weeks after treatment	Right knee	Negative rank	13	9.42	122.50	-1.836	0.066
		Positive rank	15	18.90	283.50		
	Left knee	Negative rank	11	8.18	90.00	-2.173	0.030
		Positive rank	15	17.40	261.00		

**Table 3:** WOMAC physical function scores before and after TSC and PRP intervention. TSC, total stromal cells; WOMAC, Western Ontario and McMaster University Osteoarthritis Index; z, test statistics. p-Value was obtained from Wilcoxon signed rank test.

WOMAC scores	Rank	N	Mean rank	Sum of ranks	z-Value	p-Value
2 weeks after treatment	Negative rank	29	16	464.00	-4.768	0.000
	Positive rank	1	1	1.00		
12 weeks after treatment	Negative rank	30	15.50	465.00	-4.785	0.000
	Positive rank	0	0.00	0.00		
24 weeks after treatment	Negative rank	30	15.50	465.00	-4.787	0.000
	Positive rank	0	0.00	0.00		

Before starting the treatment and after two weeks of treatment, there was no significant difference between TSC and control in terms of VAS pain, WOMAC physical function, and medial femoral cartilage thickness as measured using the Mann-Whitney U-test, though they were improved over time. The median VAS pain intensity in both knees significantly decreased after two and 12 weeks of treatment compared to before. In week 2, z scores were -4.843 and -4.832 for the left and right knee (p=0.000), respectively; after 12 weeks of post-treatment, the scores were -4.732, and z=-4.749 (p=0.000) in the left and the right knee, respectively, compared to that before treatment. At 24 weeks of treatment, the z score was -4.646 and -4.655 for the left and right knee, respectively, compared to that before treatment (p=0.000) (Table 4). 

**Table 4:** Comparison of WOMAC physical function scores between TSC and control groups before and after treatment TSC, total stromal cells; WOMAC, Western Ontario and McMaster University Osteoarthritis Index; U, test statistics. p-Value was obtained from Mann-Whitney U-test.

WOMAC	TSC group (n=15)	Control group (n=15)	U	p-Value
Before treatment	40	38	136.00	0.345
2 weeks after treatment	15	14	98.00	0.567
12 weeks after treatment	11	14	28.00	0.000
24 weeks after treatment	6	16	5.00	0.000

Besides, median WOMAC physical functioning of the knee joint was significantly improved over the periods (Table 3); at 2, 12, and 24 weeks, the z-score was z=-4.768 (p=0.000); z=-4.785 (p=0.000); and z = -4.787 (p=0.000), respectively, compared to the pretreatment scores. All the respondents of the TSC group had mild functional limitations; however, 45% (7/15) of the controls had moderate functional limitations. In the TSC group, the improvement of the WOMAC score was sustained. The changes in the mean MFC thickness were not significantly improved over the period in the right knees (z=-1.836, p=0.66); however, in the left knee after 24 weeks, there was a significant change in cartilage thickness (z=-2.173, p=0.030) (Table 5).

**Table 5:** Comparison of VAS pain and MFC thickness between TSC and control groups MFC, medial femoral condylar cartilage thickness; TSC, total stromal cells; U, test statistics; VAS, visual analog scale; WOMAC, Western Ontario and McMaster University Osteoarthritis Index. p-Value was obtained from Mann-Whitney U-test.

Particulars			Median		U	p-Value
			TSC group (n=15)	Control group (n=15)		
VAS (0-10 cm) pain	Before treatment	Right knee	6	6	107.50	0.838
		Left knee	6	6	126.50	0.567
	2 weeks after treatment	Right knee	2	2	107.00	0.838
		Left knee	2	2	107.00	0.838
	12 weeks after treatment	Right knee	2	4	24.50	0.000
		Left knee	2	4	22.50	0.000
	24 weeks after treatment	Right knee	2	4	3.0	0.000
		Left knee	2	4	3.0	0.000
MFC thickness	Before treatment	Right knee	1.93	1.93	93.50	0.436
		Left knee	1.87	1.92	66.50	0.056
	24 weeks after treatment	Right knee	2.02	1.90	175.00	0.009
		Left knee	2.02	1.90	130.00	0.016

After 12 and 24 weeks, the VAS pain scores difference of right and left knee joints between TSC and control differed statistically significantly (after 12 weeks, right, U=24.50, p=0.00; left 22.50, p=0.00, two-tailed; after 24 weeks, right U=3.0, p=0.00, left, U=3.0, p=0.00). During the periods, WOMAC physical function scores differences between the study groups were significant, and they were U=28.00 (p=0.000 two-tailed) and U=5.00 (p=0.000 two-tailed), at 12 and 24 weeks, respectively (Table 5). In addition, MFC thickness differences between TSC and control groups were statistically significant after 24 weeks, and they were U=175.00 (p=0.009 two-tailed) and U= 130.00 (p=0.016 two-tailed), respectively, for right and left knee (Table 5).

## Discussion

Multipotent MSCs derived from adipose tissue and blood, including umbilical cord blood and Wharton's jelly, have immune-modulatory, reparative, and anti-inflammatory potential and effectively heal the degenerated cartilage in OA [10,11]. It was believed that engraftment of the damaged cartilage with MSC starts healing; however, the effect proved to be associated with chondroprotective micro-ribonucleic acid (miRNA) from MSC-derived exosomes inhibiting Wnt-signaling (Wnt ligands are cysteine-rich proteins) and inducing articular cartilage regeneration [10]. 

Autologous MSCs have been safe [10,11], but the risk of tumorigenesis, disease transmission, and host immune rejection with allogeneic MSCs is still a concern [21]. Donor-site morbidity to harvest autologous MSCs would be negative compared with allogeneic MSCs [12]. Autologous MSCs should not be recommended for genetic disorders [14]. In the present study, we observed and compared VAS pain, WOMAC physical function, and US-depicted cartilage thickness between groups and pre- and post-intervention scores following autologous IA-TSC and PRP injections in knee OA. 

In a previous clinical trial, Orozco et al. treated 12 knees with OA with autologous IA-BMMSC (40×10^6^ cells) that exhibited rapid and progressive improvement of algo-functional indices (VAS pain, WOMAC, Lequesne scores) at one-year follow-up [22]; improvement was maintained even at two years [22], and there were no severe adverse events (AEs). Allogeneic MSCs have similar clinical outcomes with a few significant AEs [12,13,22,23]; however, the safety concern of allogeneic MSC therapy should be addressed in future research.

ADMSC and/or TSC seemed safe and more promising than BMMSC in clinical practice. Human adipose tissue derived by liposuction can yield adipogenic, chondrogenic, myogenic, and osteogenic cells in vitro in the presence of lineage-specific induction factors, a source of multipotent stem cells, and could be a suitable alternative source to BMMSC [24]. In a recent preclinical study with human adipose tissue, ADMSC was believed to show more promise over BMMSC in promoting neovascularization in limb ischemia, higher resistance to hypoxia-induced apoptosis, and oxidative stress-induced senescence, more potent pro-angiogenic activity, and higher expression of octamer-binding transcription factor 4 (Oct4) and VEGF [25]. Clinical research proved that autologous BMMSCs and ADMSC therapy are safe and cause an improvement in pain, physical function, patient-reported outcome measures (PROMs), and radio imaging scores at follow-up with no severe AEs [11]. 

MSCs combined with PRP and HA work in OA knee [26,27]. IA injection of infra-patellar fat pad-derived MSC (a mean of 1.18×10^6^-2.7×10^6^ stem cells) combined with PRP and arthroscopic debridement improved mean WOMAC, Lysholm, Tegner activity scale, VAS pain, and magnetic resonance imaging (MRI) scores in knee OA [26]. Furthermore, full-thickness injured knee cartilage treated with HA-MSC (ADMSC or BMMMC) yielded good to excellent clinical outcomes at long-term follow-up, irrespective of the extent of the lesion, mostly in younger individuals [27]. 

How frequently to inject? Some believe several injections can bring positive outcomes, while some support only a single injection [11,28,29]. In an RCT with symptomatic knee OA, ADMSC injected twice three weeks apart caused significant improvement of WOMAC, VAS pain, short form (SF-36) scores, and MRI-depicted cartilage volume over HA group at six- and 12-month follow-up. AEs were comparable between the two groups. Joint infection was documented in the HA group [28]. In advanced OA knee, a single IA injection of BMMSCs improved pain, WOMAC score, joint ROM, cartilage catabolic biomarkers, MRI synovitis scores, and quality of life, as seen in phase I/IIa trial [11]. In the present study, knee pain and physical function improved earlier than US-depicted articular cartilage thickness following a single shot of IA-TSC, as also depicted in a study by Lee et al. with a single shot of IA autologous AD-MSCs performed at the outpatient door clinic [29]; WOMAC stiffness and ROM but not MRI-depicted (p=.5803) cartilage defect improved significantly at six months [29]. 

ADMSC-mediated effects in OA were also reported to depend on cell counts. Jo et al. depicted the dose-dependent impact and safety concerns of ADMSC in knee OA. The higher the cell count, the higher the efficacy in improving pain, WOMAC score, and cartilage defect over medial TFJ based on radiology, arthroscopy, and histological assessments [13]. The preclinical study depicts the chondroprotective effects of extracorporeal shockwave therapy (ESWT), and the autologous IA-ADMSC combination is seen as superior over the ESWT-human umbilical cord WJMSC combination [30]; however, we still lack RCT considering the large sample size; no clinical study, including the present one, tested the efficacy of ESWT-IA-ADMSC combination [11].

MSC improves MRI-depicted poor cartilage areas (poor cartilage index, PCI) [22,23]. Orozco et al. published a comprehensive report with a two-year follow-up outcome and found an overall improvement in PCI compared to baseline (p<0.001) [23]. Serial MRI examinations explored the gradual regeneration of articular cartilage in the femoral and tibial condyles with reduced cartilage defects in femoral and tibial condyles, but not in the patella following MSC [13]; at six months, cartilage volume was seen to increase in the MFC and tibial condyles in the high-dose group, but not the cartilage defect [13]. Second-look arthroscopy also depicted macroscopic regenerated cartilage on the articular cartilages [13]. Histology revealed articular cartilage with a thick, glossy white matrix and smooth surface with the well-integrated subchondral bone. In the middle and deep zones, type-II collagen-positive hyaline-like cartilage was demonstrated; in contrast, type I-positive collagen fibrocartilage was identified in the superficial and the upper half [13]. Chondrocytes were flattened in the superficial zone and round in the middle and deep zones, similar to those in the deep zone of hyaline cartilage [13]. However, there was no improvement in the KL joint space width, mechanical axis, and anatomical axis [13]. In the present study, we depicted cartilage thickness change following TSC and PRP interventions with the US; we did not perform MRI, arthroscopy, or histology. However, further investigation is required to prove the clinical usefulness of diagnostic ultrasound in monitoring treatment outcomes, including measuring articular cartilage thickness following MSC.

Study limitations

This study was an RCT with a limited number of patients followed up only for a limited time frame. We did not evaluate the dose-dependent effects of ADMSC on different stages of articular cartilage degeneration.

## Conclusions

An IA autologous adipose-tissue-derived stromal cell provides symptomatic, functional, and US-defined articular cartilage thickness improvement in knee OA without any significant AEs. Further study with many patients over an extended period could be more informative. 
